# Nursing Home Factors and Their Impact on COVID-19 Cases: A Study of Wisconsin State

**DOI:** 10.1093/geroni/igab046.3197

**Published:** 2021-12-17

**Authors:** Mohammed Abahussain, Priya Nambisan, Colleen Galambos, Bo Zhang, Elizabeth Bukowy

**Affiliations:** 1 University of Wisconsin- Milwaukee, Wauwatosa, Wisconsin, United States; 2 University of Wisconsin- Milwaukee, Milwaukee, Wisconsin, United States; 3 Medical College of Wisconsin, Wauwatosa, Wisconsin, United States

## Abstract

COVID-19 has been devastating for Nursing Homes (NHs). The concentration of older adults with underlying chronic conditions inevitably made the setting highly vulnerable leading to high rates of mortality for residents. However, some nursing homes fared better than others. This study examines several quality measures and organizational factors to understand whether these factors are associated with COVID-19 cases in Wisconsin. We combined three datasets from Centers for Medicare & Medicaid Services (CMS) – the Star Rating dataset, Provider Information dataset and COVID-19 Nursing Home dataset. Data used is from the period of Jan 1 – Oct 25, 2020 for the state of Wisconsin. The analysis includes 331 free-standing NHs with no missing values from the data sets. The variables used were self-reported information on nursing home ratings, staff shortage, staff reported hours, occupancy rate, number of beds and ownership. Of the 331 NHs examined, shortages were reported of 25.4%, 31.1%, 3.2% and 15.6% of licensed nurse staff (25.4%), nurse aides (31.1%), clinical staff, (3.2%) and other staff (15.6%) Additionally, there was a significant (p<.05) positive correlation between number of beds and COVID-19 cases, and there was no statistically significant association between occupancy rate and COVID-19 cases. NHs with better star ratings were also found to have less COVID-19 cases. Interestingly, private NHs had significantly higher COVID-19 cases than for-profit and government owned NHs, a finding that is congruent with other studies in this area. Recommendations for practice will be discussed.

